# Effects of Machining Parameters and Tool Reconditioning on Cutting Force, Tool Wear, Surface Roughness and Burr Formation in Nickel-Based Alloy Milling

**DOI:** 10.3390/ma16227140

**Published:** 2023-11-13

**Authors:** Gábor Kónya, Zsolt F. Kovács

**Affiliations:** Department of Innovative Vehicles and Materials, GAMF Faculty of Engineering and Computer Science, John von Neumann University, Izsáki St. 10, H-6000 Kecskemét, Hungary; kovacs.zsolt@nje.hu

**Keywords:** surface roughness, trochoidal strategy, tool wear, nickel-based superalloy, slot milling, new and reconditioned tools, cutting force, burr formation

## Abstract

Nickel-based superalloys are among the most difficult materials to machine because they have high thermal strength, they are prone to hardening, carbides severely abrade the tool, and they have very poor thermal conductivity. Slot milling is a specific issue as it is characterized by rapid tool wear and frequent tool breakages. This is why reconditioned tools are frequently employed in industrial environments, as they can considerably decrease the expenses associated with tools. The chosen machining strategy also plays a crucial role in the tool’s lifespan and the quality of the machined surface, making it essential to select the appropriate strategy. Hence, the authors have opted for two conventional trochoidal strategies, namely the circular and swinging toolpath, along with a contemporary toolpath known as the Autodesk Inventor HSM Adaptive strategy. The authors investigated the effects of technological parameters and toolpaths on cutting forces, tool wear, surface roughness and burr formation on machined edges. The results show that lower cutting parameters and adaptive strategies lead to the smallest tool loads, tool wear, the best quality of surface roughness and burr formation on machined edges.

## 1. Introduction

**Figure 1:**
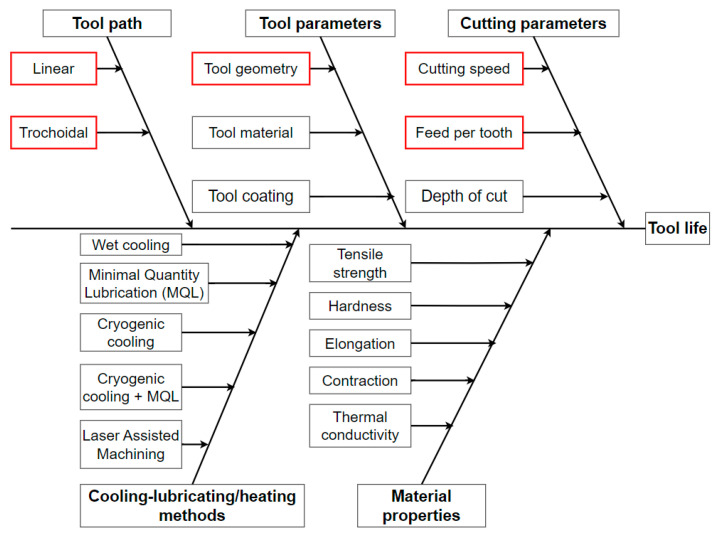
Factors influencing tool life.

Nickel-based superalloys are among the most difficult-to-machine materials due to their high tensile strength and hardness at high temperature, poor thermal conductivity and low elongation at break [1,2]. Because of these mechanical and physical properties, as illustrated in Figure 1, the machinability of these materials is low, as increased thermal expansion (1000 °C) and huge tool loading are observed during the machining process, resulting in significant cutting force and vibration [3,4], and, as a result, rapid wear of cutting tools and frequent breakages [5,6]. Tool wear is a multifaceted phenomenon arising from the interplay of both mechanical (abrasion) and chemical (diffusion) interactions between the cutting tool and the workpiece during machining processes. In the context of machining Ni-based superalloys, various forms of tool wear, such as mechanical wear, adhesive wear, diffusion wear and oxidation wear, become significantly more pronounced and problematic [7,8]. In addition, very strict tolerances must be observed with respect to the geometry of the components, which is greatly affected by tool wear [9,10,11]. A high content of metal carbides (MC, M_23_C_6_) in raw material further increases tool wear [12]. Due to these properties, high technological parameters cannot be achieved with metal carbide cutting tools, unlike with ceramic tools, where cutting speeds of up to 1000 m/min must be achieved [13].

Some parameters influence cutting tool life. Several researchers worked on investigating the effects of cooling–lubricating methods, such as wet cooling [1,14], minimum quantity lubrication (MQL) [15,16], cryogenic cooling [1,17], cryogenic cooling and MQL combination [18,19] and laser-assisted machining [20,21] in the case of machining nickel-based superalloys. However, even for these raw materials, there is considerable variation in mechanical and physical properties and clear trends cannot always be drawn, so it is worth examining the effect of these cooling–lubricating methods on all types of raw materials. Among these, the effects of linear and different types of trochoidal strategies, tool geometry, especially that of new and renovated cutting tools, and cutting parameters on the cutting force, tool wear, resulting surface roughness of the bottom of the slots and burr formation will be investigated.

Kun et al. [22] investigated the effect of cutting parameters on tool wear during the milling of a GTD-111-type nickel-based superalloy using a PVD-coated carbide indexable end mill. They found that a 10 m/min cutting speed and 0.03 mm/tooth resulted in the smallest tool wear. In the case of the other parameter combinations, the cutting inserts could not withstand the 130 mm machining length [22]. Jiang et al. (2023), in their studies, milled a GH4169-type nickel-based superalloy with a tungsten steel cutting tool with different technological parameters. They changed the cutting speed, feed per tooth and radial depth of cut and investigated their effect on cutting force and resulting surface roughness under minimal quantity lubrication. They found that a higher cutting speed and smaller feed per tooth and radial depth of cut cause less surface roughness; however, if these parameters are increased, the cutting force will also be increased [23].

There are two main types of slot milling processes, linear and trochoidal milling. Basically, linear milling has gained popularity in the industry because it is easy to program and achieves a high material removal rate (MMR). However, this leads to high cutting forces and vibration, which can cause tool failure, and the 180° contact angle makes chip removal difficult. These problems make its applicability in machining difficult-to-machine/cut metal alloys questionable, and various toolpath slotting techniques have been introduced [24,25]. Trochoidal milling is defined as a type of slot milling where the slot width must be at least 15% greater than the diameter of the tool used, the step size must be between 2 and 25% of the tool diameter, and the radial depth of cut must be no deeper than twice the tool diameter [26]. In the case of these toolpaths, the circular sections are connected by straight sections or by a continuous curve, which is constructed using a mathematical function [27]. This is the basis for various motion cycle algorithms that try to keep the contact angle constant during machining [28,29]. Consequently, the tool load can be reduced, thus reducing the rate of tool wear. Due to the reduced cutting forces, the cutting speed can be increased, thus improving the surface roughness. By reducing the contact angle, the cutting temperature is reduced, and chip flow is improved [30].

Some papers investigate the effects of cutting tool geometry, raw material and the coating of tool on the tool life and resulting surface roughness [31,32,33]. Kónya et al. (2023) first investigated the performance of new and renovated cutting tools in terms of tool loading, tool wear and surface roughness [34]. The first step in reconditioning coated cutting tools is tool grinding, where the original geometry is restored. This is followed by edge preparation, where the cutting edge is shaped according to the application, for example, by polishing. This is followed by a thorough cleaning and then coating with the desired coating. The result is that tool reconditioning has a large impact on the machining process, but the extent of this impact cannot be predicted. Such tests have great importance, as many industries use reconditioned tools, as the cost of reconditioning is one fifth of the cost of buying a new tool. In this paper, the authors investigated the effect of technological parameters on cutting force and resulting surface roughness in the case of new and renovated tools, and based on the results of this experiment row, the authors validated the previous research [35] work.

## 2. Methodology

The experimental setup for the milling experiments is presented, which includes the machining center, the device of force measurement and the cutting tool. Furthermore, the material, the difference between the tool geometry of the new and renovated tools, the technological parameters and the toolpaths are presented.

### 2.1. Experimental Setup

**Figure 2:**
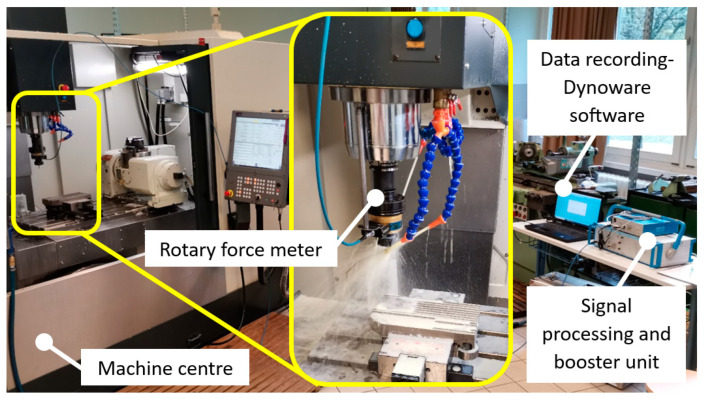
Experimental setup.

Hard milling can subject a machining center to significant stress, necessitating the use of a robust and highly rigid machine. Considering this, the NCT-EmL 850D (NCT Ipari Elektronikai Zrt., Budapest, Hungary) was selected for the experiment. The experimental setup is shown in Figure 2. The authors utilized a KISTER 9125A24 rotary force meter in conjunction with a KISTER 5327A signal booster unit and a KISTLER 5697 signal processing unit to measure the cutting force *F*_z_ component. The obtained results were recorded using DynoWare software (Version 3.2.5.0) and analyzed using OriginPro 2021 software.

### 2.2. Material

In this study, the authors utilized a Rene108-type nickel-based superalloy as the material for the workpiece. The chemical composition of this material is shown in Table 1, and the mechanical and physical properties are shown in Table 2 and Table 3.

### 2.3. Cutting Tool

For slot milling, Walter Proto max^TM^_ST_ H4038217-8-1-type solid carbide end mills were used with TiN and ZrN coatings. The tools had a diameter of 8 mm and 4 edges [35]. In this research, 13 new and 13 reconditioned tools were used, with the same technological parameters at a 1-1 ratio.

**Figure 3:**
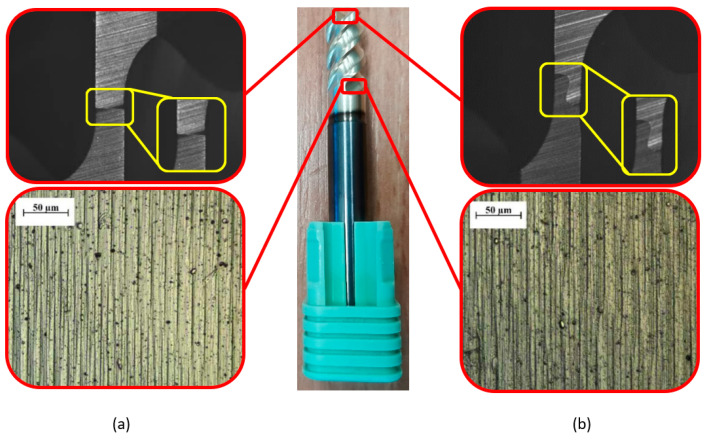
Comparison of the tool edge and working part of (**a**) new and (**b**) renovated tools.

The new and reconditioned tools were examined using a Mitutoyo Quick Vision Elf Pro microscope. Figure 3 illustrates that there was a noticeable difference in the edge geometry of the refurbished tool compared to the new one. It can be seen that the cross-edge was completely changed in the case of the renovated tool, which suggests that the original geometry could not be reproduced. It can also be observed that the grinding marks were deeper and not as even as those made with the new tool; thus, the coating quality was not as good, which had a strong influence on the friction conditions and chip separation.

### 2.4. Applied Technological Parameters for 1st Experiment Row

**Figure 4:**
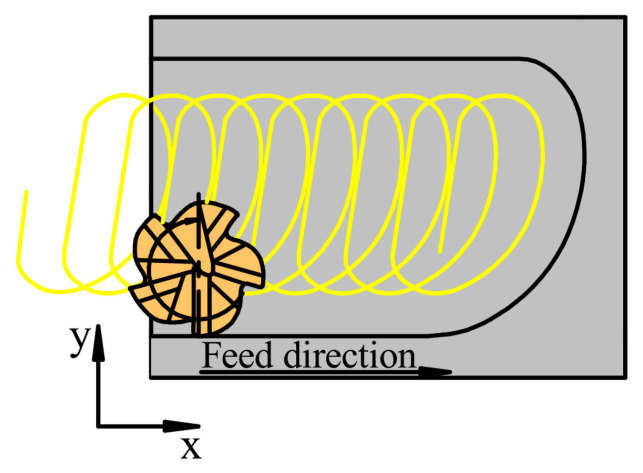
Adam Jacso’s trochoidal strategy.

The authors investigated in the first experiment row the effects of cutting speed, feed per tooth and the kind of tool used on cutting forces and the surface roughness of the bottom of the slots after milling with Adam Jacso’s trochoidal strategy, which is illustrated in Figure 4. In the experiments, flood cooling was used, and the axial depth of cut was *a*_p_ = 4 mm. Each slot was 12 × 12 × 4 mm. Cutting parameters and their levels are shown in Table 4, and the details of the experimental trials are shown in Table 5.

### 2.5. Applied Technological Parameters and Trochoidal Strategies for 2nd Experiment Row

In the second experiment, the authors investigated the effect of strategies and the kind of tool used on cutting forces and the surface roughness of the bottom of slots. The technological parameters applied for the experiments were derived from the 1st experiment row. The cutting speed was set at *v*_c_ = 25 m/min, the feed per tooth at *f*_z_ = 0.03 mm/tooth and the radial depth of cut at *a*_e_ = 0.2 mm and 0.1 mm when tool milling in the opposite direction in the case of swinging strategy. The axial depth of cut was increased to *a*_p_ = 8 mm, because this is the depth at which the industry mills slots with similar technological parameters. Each slot was 12 × 12 × 8 mm.

**Figure 5:**
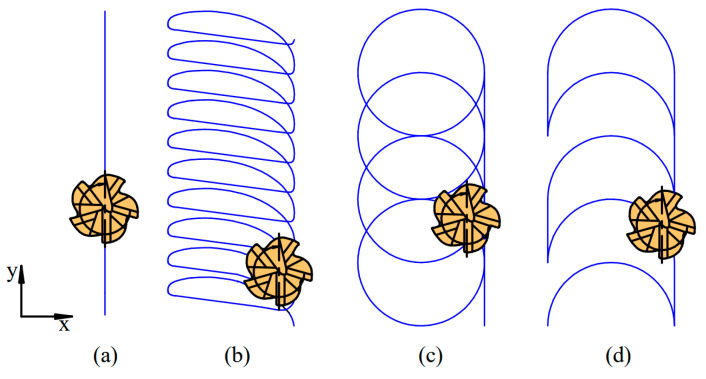
Applied strategies in comparison: (**a**) linear, (**b**) adaptive, (**c**) circular and (**d**) swinging.

The authors selected three trochoidal toolpaths for the study because these are easily programable in the industry: (a) the adaptive strategy using Autodesk Inventor HSM, (b) a circular strategy and (c) the swinging strategy developed by Szalóki et al. [26]. The specific trochoidal strategies employed are illustrated in Figure 5. Additionally, flood cooling was implemented for all slot machining processes.

## 3. Results

### 3.1. Results from 1st Experiment

#### 3.1.1. Cutting Force

**Figure 6:**
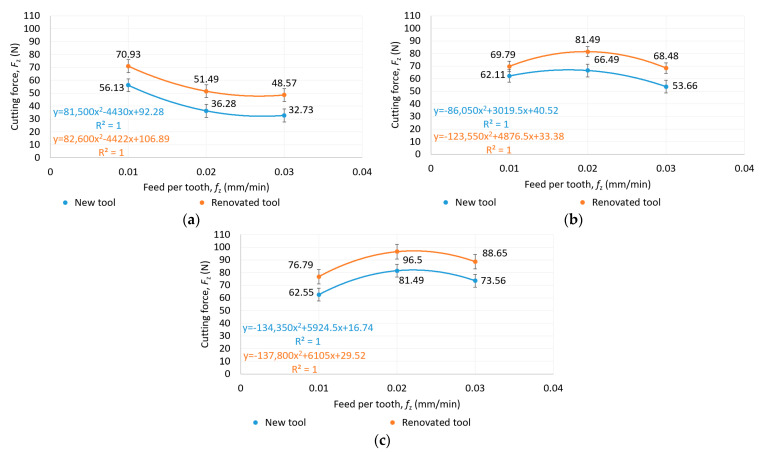
Cutting force as a function of feed per tooth in the case of (**a**) 25 m/min, (**b**) 50 m/min and (**c**) 75 m/min cutting speed for new and reconditioned tools.

**Figure 7:**
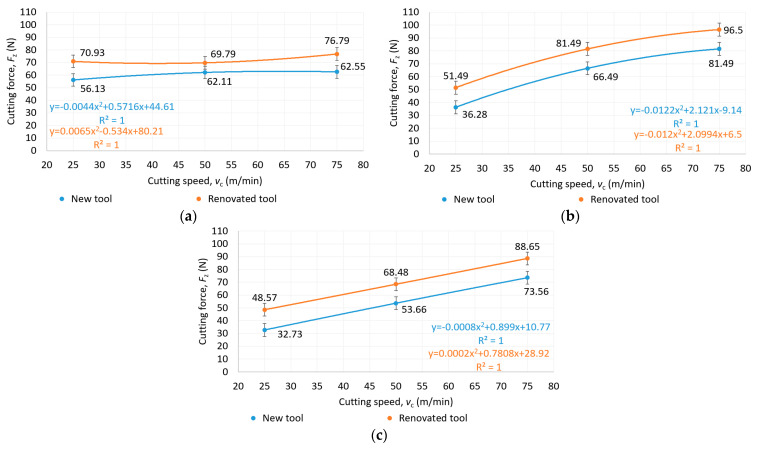
Cutting force as a function of cutting speed in the case of (**a**) 0.01 mm/tooth, (**b**) 0.02 mm/tooth and (**c**) 0.03 mm/tooth feed per tooth for new and reconditioned tools.

**Figure 8:**
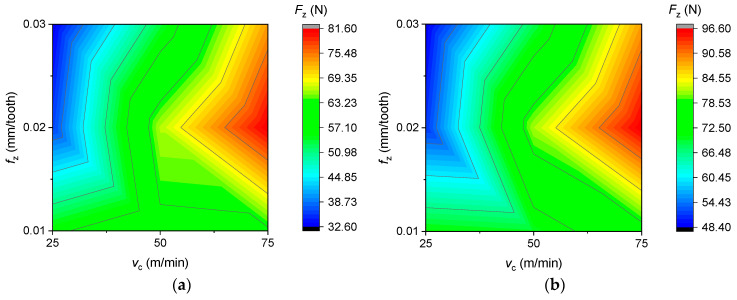
Cutting force as a function of cutting speed and feed per tooth in the case of (**a**) new and (**b**) reconditioned tools.

The cutting force as a function of feed rate and cutting speed for new and reconditioned tools is shown in Figure 6, Figure 7 and Figure 8. It was observed that in all cases, the renovated tools produced higher cutting forces than the new tools. There was no clear trend in the increase in cutting forces as a function of feed per tooth, since at a cutting speed of 25 m/min, the cutting force decreased continuously as a function of feed per tooth, while at a cutting speed of 50 and 75 m/min, a maximum value was seen at 0.03 mm/tooth feed per tooth.

As illustrated in Figure 8, as the cutting speed increased, the cutting force also increased proportionally, a trend which indicates that it has a greater effect on tool load than the feed per tooth. The lowest cutting force was achieved at a cutting speed of 25 m/min and a feed per tooth of 0.03 mm/tooth, as shown in Figure 8.

#### 3.1.2. Surface Roughness

**Figure 9:**
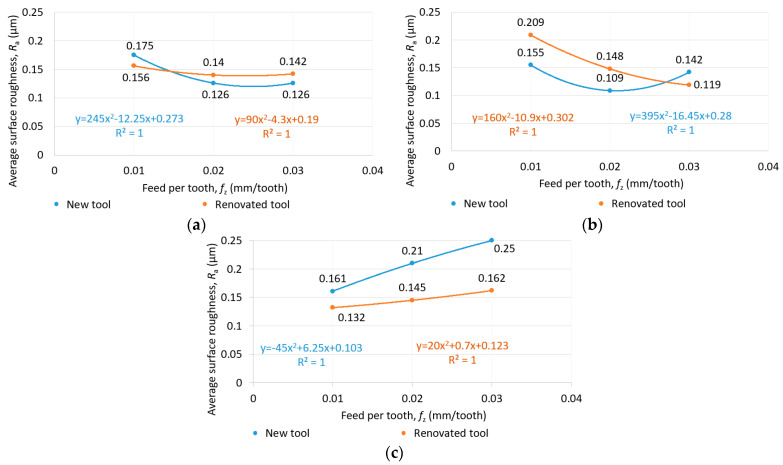
Average surface roughness as a function of feed per tooth in the case of (**a**) 25 m/min, (**b**) 50 m/min and (**c**) 75 m/min cutting speed for new and reconditioned tools.

**Figure 10:**
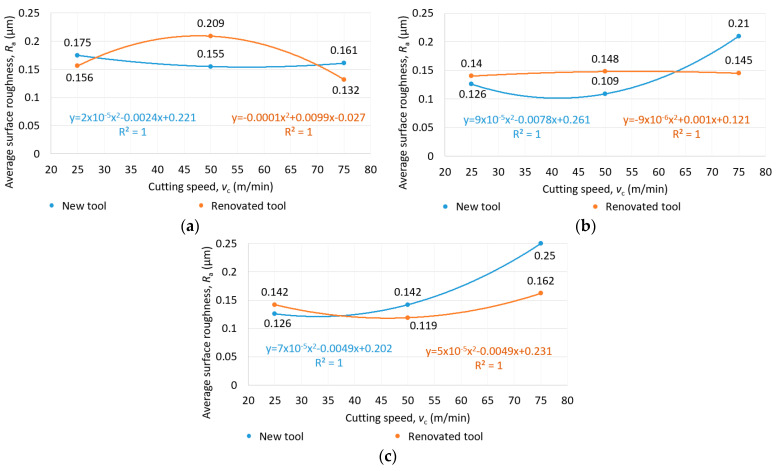
Average surface roughness as a function of cutting speed in the case of (**a**) 0.01 mm/tooth, (**b**) 0.02 mm/tooth and (**c**) 0.03 mm/tooth feed per tooth for new and reconditioned tools.

**Figure 11:**
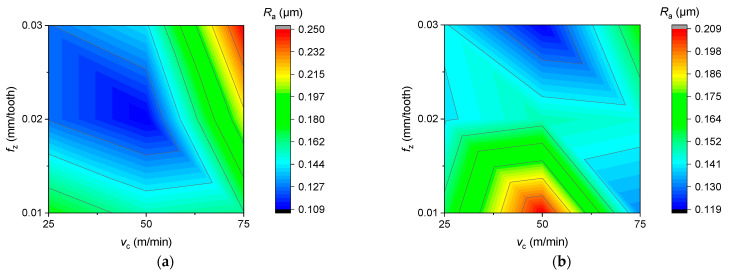
Average surface roughness as a function of cutting speed and feed per tooth in the case of (**a**) new and (**b**) reconditioned tools.

The average surface roughness (*R*_a_) as a function of feed per tooth and cutting speed for new and reconditioned tools is shown in Figure 9, Figure 10 and Figure 11. In terms of average surface roughness, there was no clear trend showing whether the new tool or the renovated tool produced a better surface. Contrary to the literature research review, in the case of Rene108, it was found that increasing the cutting speed increased the surface roughness. The effect of cutting speed was greater than that of the feed per tooth on average surface roughness. The smallest average surface roughness was achieved at a 25 m/min cutting speed and 0.03 mm/tooth feed per tooth, as shown in Figure 11.

#### 3.1.3. The Goodness of Tool Reconditioning

**Figure 12:**
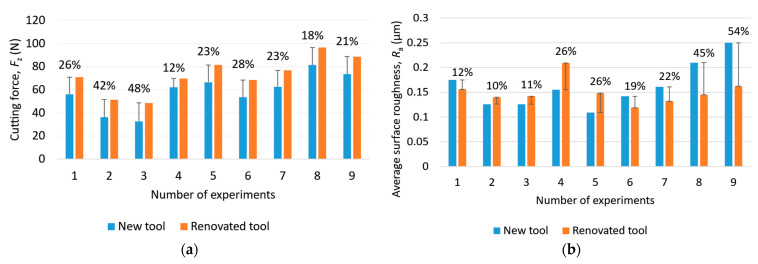
The goodness of tool reconditioning in the case of (**a**) cutting force and (**b**) average surface roughness.

The goodness of tool reconditioning for cutting force and average surface roughness is illustrated in Figure 12. It can be seen that the quality of the tool reconditioning had a very large effect on the cutting force, as a difference of 12–48% in cutting force was measured between the new tool and the resharpened tool for the same process parameters. This is a very large variance, which makes the machining process unstable in automated production. The differences in average surface roughness had a similar variance, between 10 and 54%, suggesting that tool reconditioning has a similarly significant effect on surface roughness as on cutting force. There were also cases where the renovated tool achieved better surface quality than the new tool. This is because the cutting edges had a larger radius. This surface roughness difference was larger in the case of the higher cutting speed.

### 3.2. Results from 2nd Experiment

#### 3.2.1. Cutting Force

**Figure 13:**
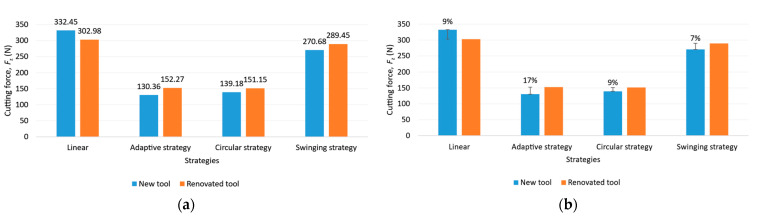
(**a**) Cutting force for each strategy; (**b**) the goodness of tool reconditioning in each strategy.

The measured cutting force for new and renovated tools for each strategy and the goodness of tool reconditioning are shown in Figure 13. The linear strategy led to the highest value of cutting force, but it is important to note that both tools were used to failure here to see how much tool load they would fail under. As can be seen, there was a 9% difference in performance between the two tools, but since the same difference was seen in the performance of the renovated tools, it is possible that another tool would have been able to bear a greater load. The lowest cutting force was observed for the adaptive strategy, followed by the circular and then the swinging strategy, where more an increase in cutting force of almost two times was observed compared to the adaptive strategy. This is due to the directional changes in the toolpath, as the strategy conducts up and down milling in one machining operation.

#### 3.2.2. Average Surface Roughness Evaluation in the Second Experiment

**Figure 14:**
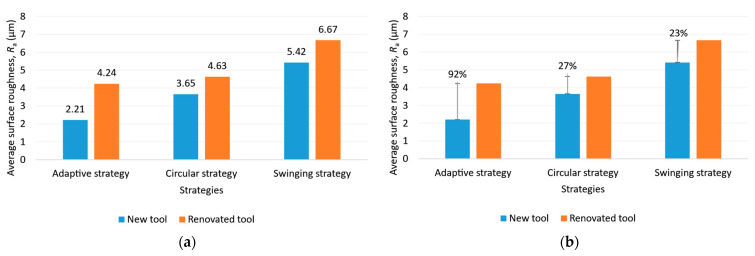
(**a**) Average surface roughness for each strategy; (**b**) the goodness of tool reconditioning for each strategy.

As illustrated in Figure 14, the best surface roughness was obtained with the adaptive strategy, followed by the circular and swinging strategy. This is due to changes in the direction of the toolpaths. It is interesting to note that in the case of the adaptive strategy, machining with the renovated tool resulted in almost twice the surface roughness compared to the surface machined with the new tool. The difference was almost the same for the other two strategies.

#### 3.2.3. Tool Wear

**Figure 15:**
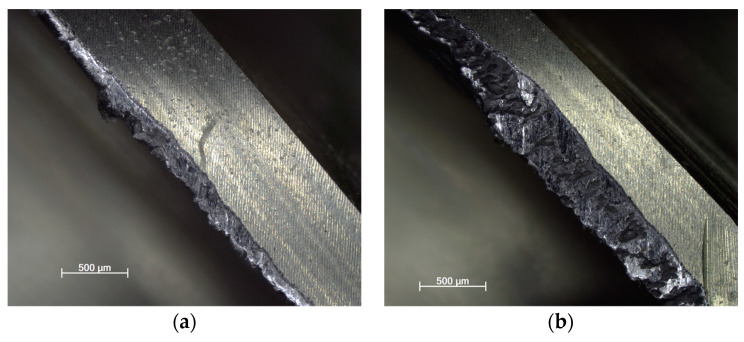
(**a**) New and (**b**) renovated tools used in adaptive strategy.

**Figure 16:**
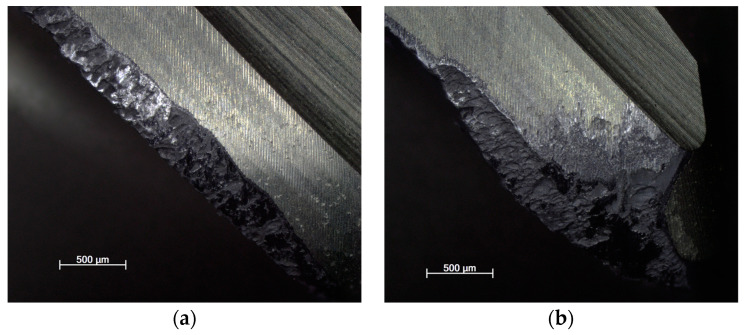
(**a**) New and (**b**) renovated tools used in circular strategy.

**Figure 17:**
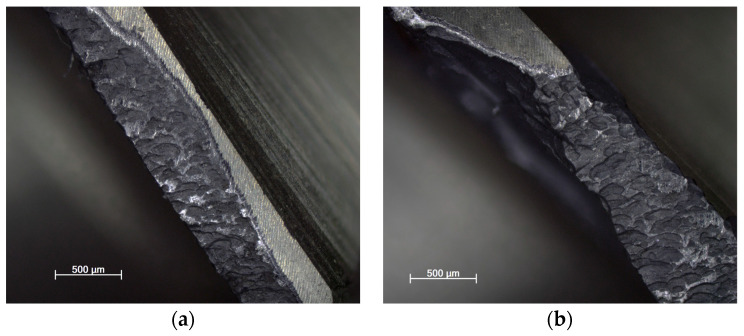
(**a**) New and (**b**) renovated tools used in swinging strategy.

The tool wear using each strategy for new and reconditioned tools is shown in Figure 15, Figure 16 and Figure 17. It can be said that in all cases, the renovated tool was more damaged. This is also because the geometry changed compared to the original, having a rougher surface due to the different grinding process, resulting in a change in the friction conditions.

Regarding the cutting force, the lowest tool wear occurred when milling with the adaptive strategy, followed by the circular and pendulum strategy. With the adaptive strategy, abrasive wear and pitting were visible. With the circular strategy, more intensive abrasive wear and pitting were seen. The tools used for the swinging strategy experienced the greatest wear and, in the case of the renovated tool, the cutting edge was torn off. Here, proper toolpath design and using toolpaths with a constant contact angle are the solution for smaller tool wear and tool load. As can be seen, the directional change in the toolpath had a great influence on tool life.

#### 3.2.4. Burr Formation on Machined Edges

**Figure 18:**
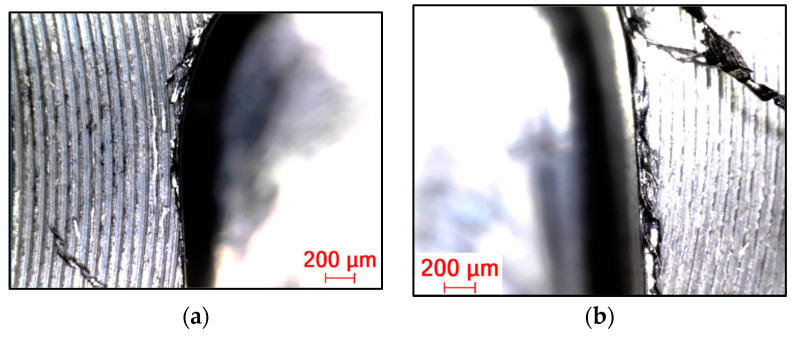
Burr in the case of machining with (**a**) new and (**b**) renovated tools used in adaptive strategy.

**Figure 19:**
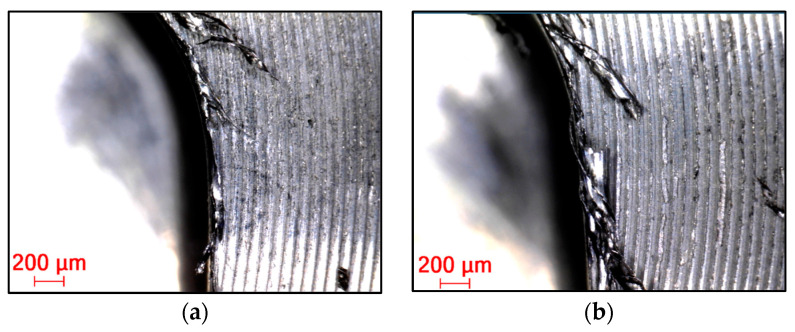
Burr in the case of machining with (**a**) new and (**b**) renovated tools used in circular strategy.

**Figure 20:**
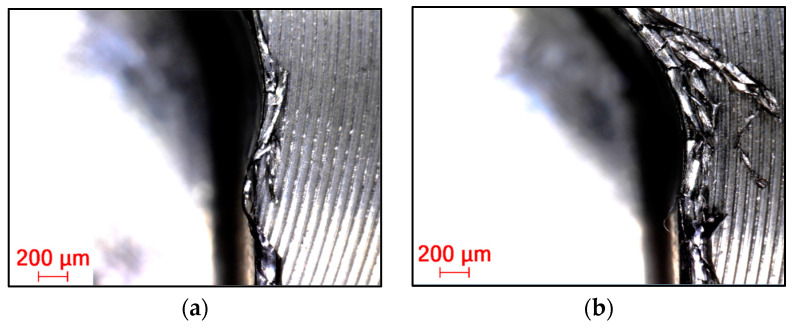
Burr in the case of machining with (**a**) new and (**b**) renovated tools used in swinging strategy.

Burrs in general can be defined as unseparated chips and are formed largely due to the cutting edge radius being larger than the undeformed chip thickness, causing a ploughing effect due to the excessive plastic flow of the material [30]. This phenomenon occurs to a lesser or greater extent in almost all cases, especially with difficult-to-machine materials. In all cases (Figure 18, Figure 19 and Figure 20), a top and cut-off burr was seen, the size of which varied depending on the strategy used. The smallest burr was achieved in the case of the adaptive strategy, while the largest was visible in the case of the swinging strategy. The magnitude of the burr was directly proportional to the magnitude of the cutting force, as Santos et al. (2023) described in their studies. According to Santos et al. (2023), as the radius of the cutting edge increases compared to the feed per tooth, the burr size increases [36]. This explains why machining with a resharpened tool resulted in a larger line for each strategy.

## 4. Conclusions

This paper examined the effects of technological parameters, tool conditions and milling strategies on the cutting force, tool wear and average surface roughness of milled slots. The authors reached the following conclusions:

- The applied technological parameters have a great influence on the cutting force and average surface roughness of milled slots.
- The applied strategies have a great effect on tool life and average surface roughness due to directional changes. It is advisable to use toolpaths that ensure a constant contact angle.
- Tool reconditioning has a great influence on tool life and average surface roughness of milled slots; however, this influence is unpredictable, as seen from the goodness of tool reconditioning.
- The applied trochoidal strategy and tool reconditioning have a great influence on burr formation on the machined edge.

## Figures and Tables

**Table 1:** Chemical composition of Rene108.

Ni (%)	C (%)	Cr (%)	Co (%)	Al (%)	Ti (%)	W (%)	Mo (%)	Ta (%)	Zr (%)	B (%)	Hf (%)
63.3	0.07	8.00	9.00	5.25	0.60	9.30	0.40	2.80	0.005	0.01	1.3

**Table 2:** Mechanical properties of Rene108.

Tensile Strength, *R*_m_ (MPa)	Elongation, *A*_5_ (%)	Contraction, *Z* (%)	Hardness, HRC
1331	8	10	42.1

**Table 3:** Physical properties of Rene108.

Density *ρ* (kg/m^3^)	Thermal Conductivity *λ* (W/mK)	Specific Heat, c (J/kgK)
8130	12.10	0.444 × 10^3^

**Table 4:** The input parameters and their levels.

Input Parameters	Levels		
Cutting speed, *v*_c_ (m/min)	25	50	75
Feed per tooth, *f*_z_ (mm/min)	0.01	0.02	0.03

**Table 5:** Experimental trials.

Exp. No.	*v*_c_ (m/min)	*f*_z_ (mm/min)
1.	25	0.01
2.	25	0.02
3.	25	0.03
4.	50	0.01
5.	50	0.02
6.	50	0.03
7.	75	0.01
8.	75	0.02
9.	75	0.03

## Data Availability

Data is contained within the article.
